# Correlation of clinical presentation with prognosis in oral squamous cell carcinoma associated with oral submucous fibrosis

**DOI:** 10.1186/s12885-025-14415-2

**Published:** 2025-06-05

**Authors:** H. Hande Alka, Gadbail Amol, Sonone Archana, K. Patil Swati, Pakhale Aayushi, N. Sharma Preethi

**Affiliations:** 1 Department and Institution, Department of Oral Pathology and Microbiology, Sharad Pawar Dental College and Hospital, Datta Meghe Institute of Higher Education & Research (Deemed to be University, Wardha, Maharashtra 442001 India; 2 Department and Institution, Department of Dentistry, Government Medical College & Hospital, Nagpur, Maharashtra India

**Keywords:** OSCC, OSMF, Clinical presentation, Prognosis, Erythroplakic, Erythro-leukoplakic, Ulcerative/endophytic, Ulcero-proliferative, Proliferative/exophytic

## Abstract

**Background:**

Oral squamous cell carcinoma **(**OSCC) associated with oral submucous fibrosis (OSMF) constitutes a clinicopathologically distinct entity in context to younger age of presentation, better histological grade of tumor differentiation, and lower risk of nodal metastasis. This study is intended to provide the basis of clinical presentation of OSCC associated with OSMF to consider a clinicopathologically unique entity compared to OSCC without OSMF.

**Methods:**

The cohort of 320 OSCC patients was divided into two groups based on the association of OSMF. Group one, OSCC without OSMF (*n* = 166), whereas Group two, OSCC associated with OSMF (*n* = 154). The varied clinical presentation were categorized as, erythroplakic, erythro-leukoplakic, ulcerative/endophytic, ulcero-proliferative, and proliferative/exophytic. The variations in clinical presentation between the OSCC without OSMF and OSCC associated with OSMF groups were assessed using one-way ANOVA and the Tukey’s HSD test.

**Results:**

Group one cohort presented with, significantly high number of cases clinically as ulcerative/endophytic 72 (43.4%) followed by ulcero-proliferative 53 (31.9%), erythro-leukoplakic 30 (18.1%), proliferative/exophytic 8 (4.8%) and erythroplakic 3 (1.8%). Whereas in Group two, significantly high number of cases clinically presented as nearly equal number of ulcero-proliferative 55 (35.7%) and proliferative/exophytic 50 (32.5%) followed by ulcerative/endophytic 25 (16.2%), erythro-leukoplakic 18 (11.7%) and erythroplakic 6 (3.9%) (*p* < 0.001).

**Conclusion:**

Significantly high number of cases of OSCC associated with OSMF clinically presented as ulcero-proliferative and proliferative/exophytic. This characteristic clinical presentation may contribute in early detection and diagnosis by providing considerable period for recognition and treatment planning and thus better prognosis in OSCC associated with OSMF cases.

## Background

The habit of chewing areca nut and smokeless tobacco is the primary cause of the high prevalence of OSCC in India [1]. The International Agency for Research on Cancer, World Health Organization, states in its monographs from 1985 to 2004 that areca nut is an independent group 1 human carcinogen [2]. Oral submucous fibrosis (OSMF), a common oral potentially malignant disease (OPMD) marked by progressive fibrosis of the oral mucosa, is caused by areca nut consumption in combination with slaked lime and other ingredients [3]. The habitual consumption of commercially prepared smokeless tobacco and areca nut products has become more widespread amongst youngsters due to easy availability, affordability and marketing strategies of the product. This has contributed to significant increase in the cases of OSMF in younger population [4]. In Southeast Asian nations malignant transformation of OSMF is highly prevalent and OSCC concomitant with OSMF is one of the most common malignancies [5].Chourasia et al. (2006) observed an incidence of 25.77% in OSCC associated with OSMF [6]. The alternative pathways of areca nut induced carcinogenesis are believed to be attributable to this clinicopathologically distinct entity of OSCC associated with OSMF [7–9].

The varied clinical presentation of OSCC is correlated with site of the lesion, period of advancement, relationship with precancerous lesions, and risk factors. Additionally, lesions are typically asymptomatic; pain and discomfort only manifest after muscles or nerves have been invaded during an advanced stage of the disease. Leukoplakia, erythroplakia, or erythro-leukoplakia that has already progressed to malignancy are few of the diverse clinical presentations. Other probable presentations include necrotic ulcers with unevenly elevated, indurated borders, broad-based exophytic masses with surfaces that can be pebbled, verrucous, or generally smooth [10].

In OSCC associated with OSMF, the clinical presentation and behavior is variable. Although exophytic proliferative lesions in the context of OSCC associated with OSMF are not very uncommon, there is a dearth of appropriate data especially from Southeast Asian nations where the prevalence of OSMF is significant. Consistent with the prevailing information, the prognosticators of OSCC associated with OSMF in context to the clinical presentation of the lesion have not been comprehensively explored [11].This study is intended to provide the basis of clinical presentation of OSCC associated with OSMF to consider a clinicopathologically unique entity compared to OSCC without OSMF.

## Methods

The study was conducted following the approval of the Institutional Ethics Committee for a retrospective analysis “(DMIMS (DU) IEC/Dec-2019/8556- dated-17-12-2019)”.Written informed consent was obtained from all patients for the use of their clinical data and archived tissue samples. Subsequently, data from patients treated between 2009 and 2015 were retrieved from the archives of the Department of Oral Pathology and Microbiology. Statistical analysis for sample size calculation has been done as follows.

OSCC = X^2^ x N x P x Q/C^2^ x (N-1) + X^2^ x P x Q.

= 3.84 × 300 × 0.5 × 0.5/ (0.05)^2^ × 299 + 3.84 × 0.5 × 0.5.

= 288/0.74 + 0.96

= 288/1.70

= 169.41 = 170

Where N = Total No. of patients sample in last three years visited to department; P is 50% proportion rate (0.5); Q= (1-P) = 0.5;C = Confidence interval of one’s choice = 0.05;X^2^ = Chi square value for 1 degree of freedom at some desired probability level i.e. 3.84.

(Actual sample included in this study = 320 with 5 year survival data)

**Fig. 1:**
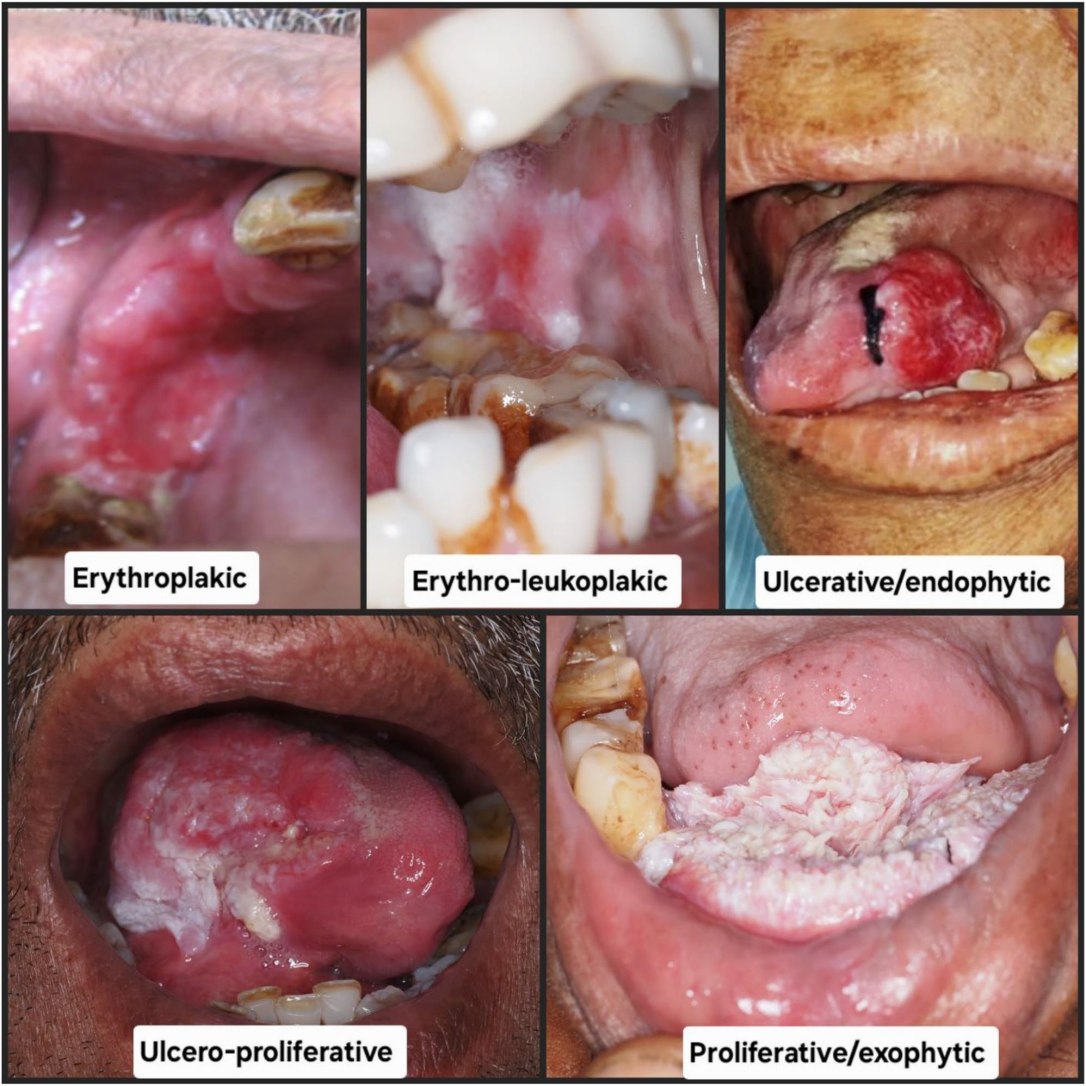
Clinical presentations of OSCC Erythroplakic-Red patches of mucosa with a high malignant potential Erythro-leukoplakic- mixed red and white lesions Ulcerative/endophytic- lesions characterized by ulceration and inward tissue growth Ulcero-proliferative- ulcers accompanied by outward proliferative growth Proliferative/exophytic- mass-forming lesions that grow outward from the surface

Thus the study population consisted of 320 surgically treated cases of OSCC each confirmed through clinical and histological diagnosis. The significant cohort of OSCC patients was divided into two groups based on the association of OSMF. Group one consists of 166 cases of OSCC without OSMF, whereas Group two consists of 154 cases of OSCC associated with OSMF. Patients with primary disease as OSCC and tumor site specific to the oral cavity are included in the study. Patients who had undergone preoperative chemotherapy or radiotherapy for multiple primary tumors, recurrent, or metastatic OSCC were excluded from the study. Age, gender, a complete history of each relevant habit with its dose and duration, as well as the location of the lesion for the entire study population, were obtained. Clinical staging of patients was performed in accordance with the “American Joint Committee of Cancer’s guidelines” for tumor node metastatic disease [12]. Additional thorough details regarding the lesion’s clinical presentation were noted. The varied clinical presentations of OSCC were categorized as follows: erythroplakic (red patches of mucosa with a high malignant potential), erythro-leukoplakic, (mixed red and white lesions), ulcerative/endophytic (lesions characterized by ulceration and inward tissue growth), ulcero-proliferative (ulcers accompanied by outward proliferative growth), and proliferative/exophytic, (mass-forming lesions that grow outward from the surface). (Fig. 1) For histopathological analysis, tissue sections stained with hematoxylin and eosin were retrieved from the department’s archives. All OSCC cases were independently graded by three oral pathologists who were blinded to the original diagnosis using the Anneroth’s grading system, a histopathological grading system that classifies tumors based on the degree of keratinization, nuclear pleomorphism, number of mitoses, pattern of invasion, stage of invasion and lymphoplasmacytic infiltration of tumor cells, into well differentiated, moderately differentiated and poorly differentiated tumor. Lymph node metastasis was evaluated through histopathological assessment of surgically dissected lymph nodes. The data for five years of disease-free survival was recorded.

## Statistical analysis

The variations in clinical presentation between the OSCC without OSMF and OSCC associated with OSMF groups were assessed using “one-way ANOVA” and the “Tukey’s HSD test”. To determine the difference in mean age between OSCC associated with OSMF and OSCC without OSMF, independent student *t* tests were employed. The gender, location, clinical TNM stage, histological grading, metastasis to the cervical lymph node, 5-year survival, and clinical presentation within OSCC associated with OSMF and OSCC without OSMF were all compared using the chi-square test. Statistical analysis of the records was accomplished using “SPSS for Windows, version 17.0”. The statistical significance level was set at “p 0.05”.

## Results

**Graph 1:**
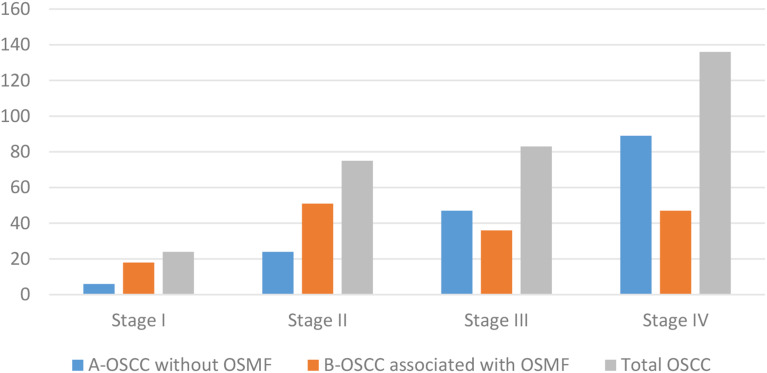
TNM Staging in OSCC cases TNM-Tumor Node Metastasis, OSCC-Oral Squamous Cell Carcinoma OSMF-Oral Submucous Fibrosis

**Graph 2:**
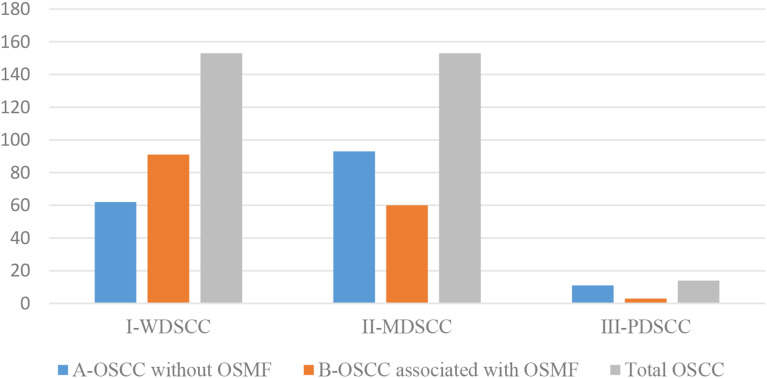
Histopathological Grading in OSCC cases OSCC-Oral Squamous Cell Carcinoma, OSMF-Oral Submucous Fibrosis WDSCC-well differentiated squamous cell carcinoma, MDSCC-moderately differentiated squamous cell carcinoma, PDSCC-poorly differentiated squamous cell carcinoma

**Graph 3:**
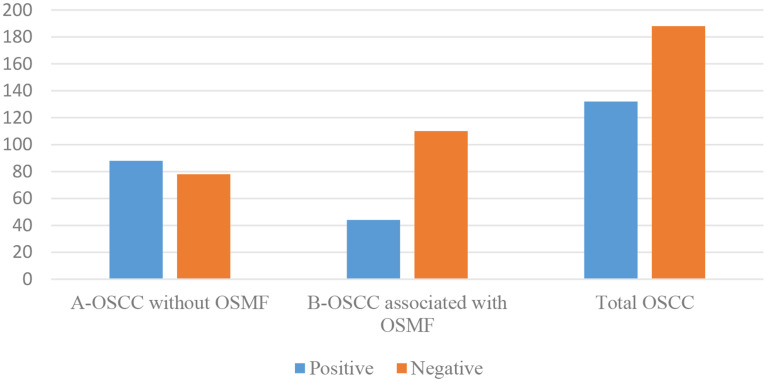
Metastasis to cervical lymph nodes in OSCC cases OSCC-Oral Squamous Cell Carcinoma, OSMF-Oral Submucous Fibrosis

**Graph 4:**
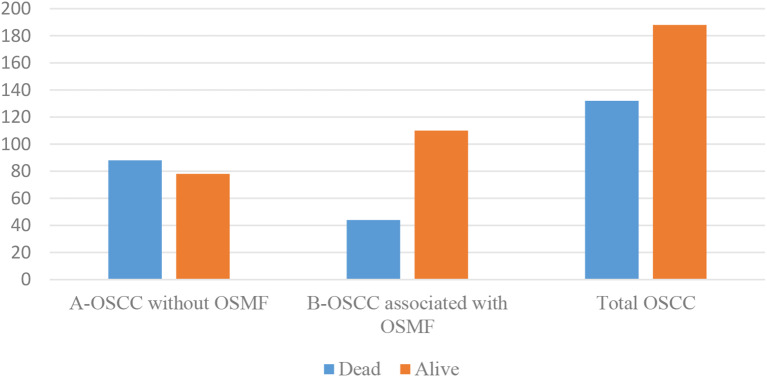
Survival status in OSCC cases OSCC-Oral Squamous Cell Carcinoma, OSMF-Oral Submucous Fibrosis

**Graph 5:**
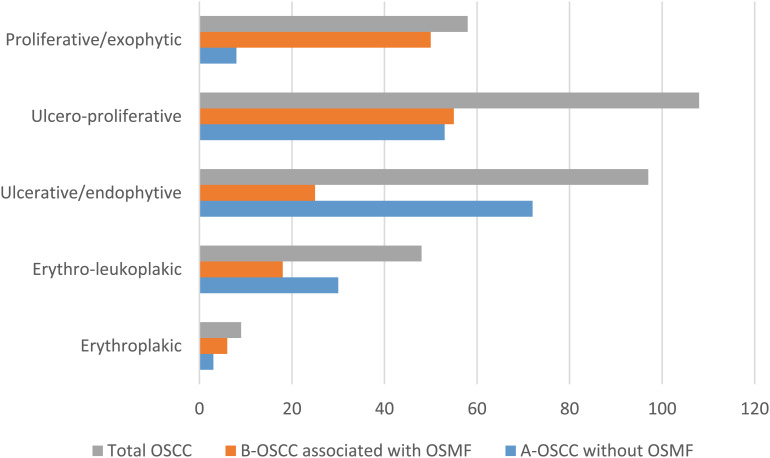
Clinical presentations in OSCC cases OSCC-Oral Squamous Cell Carcinoma, OSMF-Oral Submucous Fibrosis

A total of 320 OSCC cases, fulfilling the inclusion and exclusion criteria were included in the study. The patients’ ages’ ranged from 20 to 81 years with the mean age of 52 ± 13.00 years. OSCC was predominantly observed in males (76.2%) with a male to female ratio of 3.21:1. The most commonly affected site was the buccal mucosa (43.8%) followed by the gingivo-buccal sulcus (36.2%), tongue (8.8%), palatal mucosa (4.4%), retro molar region (4.4%) and labial mucosa (2.5%). Clinically, the majority of OSCC cases were diagnosed at TNM stage IV (42.5%) followed by stage III (25.9%), stage II (23.4%) and stage I (7.5%) (Graph 1). The histopathological diagnosis of 50% cases of OSCC was well differentiated squamous cell carcinoma (WDSCC, 47.8%) and moderately differentiated squamous cell carcinoma (MDSCC, 47.8%), whereas poorly differentiated squamous cell carcinoma (PDSCC) was reported in only 4.4% of cases (Graph 2). Metastasis to cervical lymph node was reported in 137 (42.8%) cases of OSCC (Graph 3). At the end of a 5-year follow-up of 320 OSCC cases, an overall survival rate of 58.8% was observed (Graph 4). Among all clinically evaluated OSCC cases, the most common presentation was the ulcero-proliferative type, observed in 33.8% of cases. This was followed by the ulcerative/endophytic (30.3%), proliferative/exophytic (18.1%), erythro-leukoplakic (15.0%), and erythroplakic in 2.8% of cases (Graph 5) (Table 1).

**Table 1:** Comparison of demographic and clinicopathological parameters between oral squamous cell carcinoma without OSMF (OSCC), and oral squamous cell carcinoma associated with oral submucous fibrosis (OSCC-OSMF) patients

Characteristics		OSCC (*n* = 166)	OSCC-OSMF (*n* = 154)	Total OSCC (*n* = 320	Student t test/ chi-square test	
Age	Mean	56.13	46.81	52	*p* < 0.001	
	SD	11.09	12.59	13.00		
	Range	30–81	20–80	20–81		
Gender	Male	120 (72.3%)	124 (80.5%)	244 (76.2%)	*p* = 0.084	
	Female	46 (27.7%))	30 (19.5%)	76 (23.8%)		
Site	BM	60 (36.1%)	80 (51.9%)	140 (43.8%)	*p* < 0.001	
	GBS	80 (48.2%)	36 (23.4%)	116 (36.2%)		
	LM	4 (2.4%)	4 (2.6%)	8 (2.5%)		
	PM	9 (5.4%)	5 (3.2%)	14 (4.4%)		
	RM	6 (3.6%)	8 (5.2%)	14 (4.4%)		
	TNG	7 (4.2%)	21 (13.6%)	28 (8.8%)		
TNM staging	Stage I	6 (3.6%)	18 (11.7%)	24 (7.5%)	*p* < 0.001	
	Stage II	24 (14.5%)	51 (33.1%)	75 (23.4%)		
	Stage III	47 (28.3%)	36 (23.4%)	83 (25.9%)		
	Stage IV	89 (53.6%)	47 (30.5%)	136 (42.5%)		
Histopathological Grading	WDSCC	62 (37.3%)	91 (59.1%)	153 (47.8%)	*p* < 0.001	
	MDSCC	93 (56.0%)	60 (39.0%)	153 (47.8%)		
	PDSCC	11 (6.6%)	3 (1.9%)	14 (4.4%)		
Metastasis to cervical lymph node	Positive	88 (53.0%)	49 (31.8%)	137 (42.8%)	*p* < 0.001	
	Negative	78 (47.0%)	105 (68.2%)	183 (57.2%)		
Survival status	Dead	88 (53.0%)	44 (28.6%)	132 (41.2%)	*p* < 0.001	
	Alive	78 (47.0%)	110 (71.4%)	188 (58.8%)		
Clinical presentation	Erythroplakic	3 (1.8%)	6 (3.9%)	9 (2.8%)	*p* < 0.001	
	Erythro-leukoplakic	30 (18.1%)	18 (11.7%)	48 (15.0%)		
	Ulcerative/endophytic	72 (43.4%)	25 (16.2%)	97 (30.3%)		
	Ulcero-proliferative	53 (31.9%)	55 (35.7%)	108 (33.8%)		
	Proliferative/exophytic	8 (4.8%)	50 (32.5%)	58 (18.1%)		

Abbreviations– BM-buccal mucosa, GBS-gingivobuccal sulcus, LM- labial mucosa, PM-palatal mucosa, RM- retromolar mucosa, TNG-tongue, TNM-Tumor Node Metastasis, WDSCC-well differentiated squamous cell carcinoma, MDSCC moderately differentiated squamous cell carcinoma, PDSCC-poorly differentiated squamous cell carcinoma

**Fig. 2:**
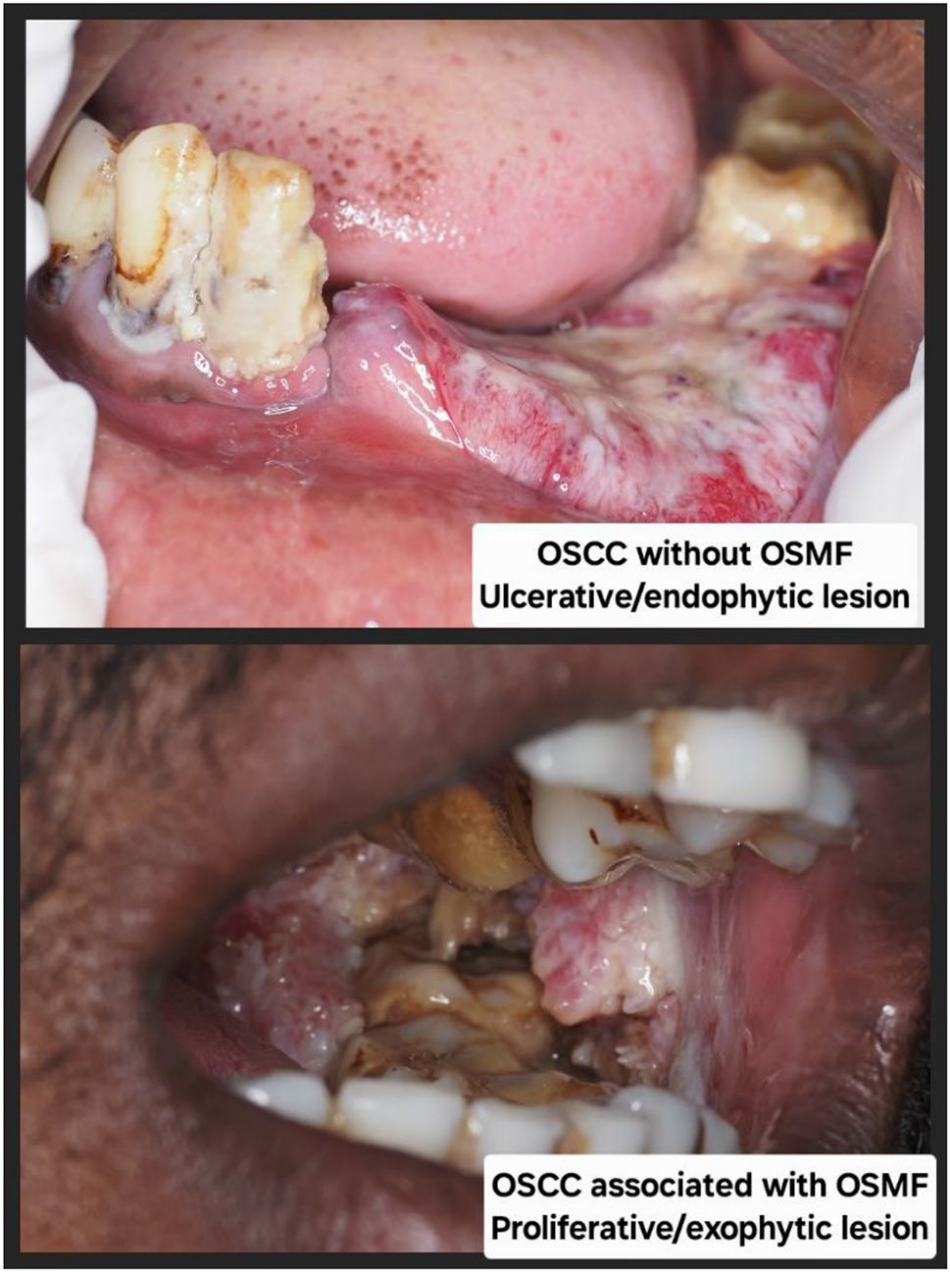
Clinical presentations of OSCC without OSMF and OSCC associated with OSMF OSCC-Oral Squamous Cell Carcinoma, OSMF-Oral Submucous Fibrosis Ulcerative/endophytic- lesions characterized by ulceration and inward tissue growth Proliferative/exophytic- mass-forming lesions that grow outward from the surface

Further the present cohort of OSCC was divided into two categories, OSCC without OSMF (Group 1) and OSCC associated with OSMF (Group 2) on the basis of clinical presentation. (Fig. 2) Group1 cohort presented with, significantly high number of cases clinically as ulcerative/endophytic 72 (43.4%) followed by ulcero-proliferative 53 (31.9%), erythro-leukoplakic 30 (18.1%), proliferative/exophytic 8 (4.8%) and erythroplakic 3 (1.8%). Whereas in Group 2, significantly high number of cases clinically presented as nearly equal number of ulcero-proliferative 55 (35.7%) and proliferative/exophytic 50 (32.5%) followed by ulcerative/endophytic 25 (16.2%), erythro-leukoplakic 18 (11.7%) and erythroplakic 6 (3.9%) (*p* < 0.001). (Table 1)

## Discussion

Even though OSCC prevalence and associated morbidity and death vary widely over the world, they are higher in areas with significant areca nut and tobacco usage. Additionally, the OSCC in the background of OSMF is one of the most prevalent carcinoma in the population of South East Asia as a result of imprudent use of tobacco and areca nut products, with the malignant transformation rate of 6% [13]. The clinical manifestation of OSCC may exhibit characteristics of the precursor lesion, which is an OPMD. Such OPMDs where the transformation into OSCC may take place include leukoplakia, erythroleukoplakia, proliferative verrucous leukoplakia, verrucous hyperplasia, oral lichen planus, and OSMF. However, there are variations in the clinical signs and symptoms amongst these lesions. The proliferative, exophytic pattern was more prevalent in OSCC associated with OSMF as compared to infiltrative and ulcero-infiltrative clinical presentation [14]. Although across European and North American Pathology practices the oral verrucous hyperplasia (OVH) is observed very rarely but is increasingly correspond to the province of South East Asia [15]. OVH in the background of OSMF is a relatively common pathology observed especially amongst habitual users of areca nut and exhibits a distinct clinicopathological characteristics [16], with a malignant transformation rate of 7 − 13% [17].

**Fig. 3:**
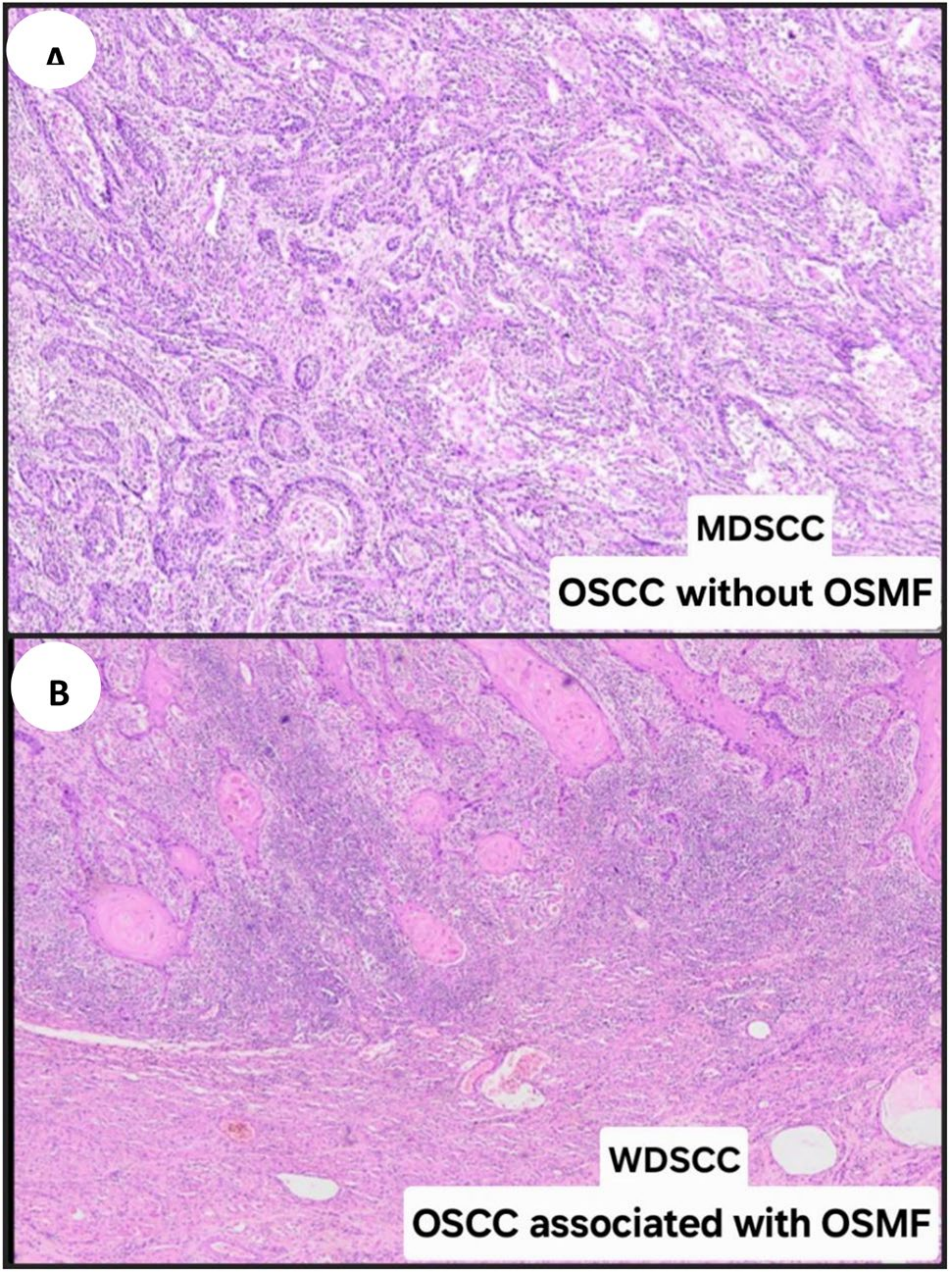
Histopathological presentation of OSCC without OSMF and OSCC associated with OSMF **A**. Presence of neoplastic cells arranged in strands and cords showing hyperchromatism, nuclear and cellular pleomorphism. No evidence of keratin pearl formation. All the features are suggestive of MDSCC (Moderately differentiated squamous cell carcinoma) in OSCC without OSMF. (Hematoxylin and Eosin staining, 100X) **B**. Presence of keratin pearls, marginated by marked inflammatory cell infiltration at the invasive tumor front. The tumor islands are bounded by collagen fiber bundles. All the features are suggestive of WDSCC (Well differentiated squamous cell carcinoma) in OSCC associated with OSMF. (Hematoxylin and Eosin staining, 100X) OSCC-Oral Squamous Cell Carcinoma, OSMF-Oral Submucous Fibrosis

The evaluation of all OSCC cases was done in terms of clinicopathological characteristics and treatment outcome. Further the differences between OSCC associated with OSMF and OSCC without OSMF in terms of clinical presentation was evaluated more comprehensively. Our organization’s prior research had illustrated that the OSCC associated with OSMF was a unique clinicopathological entity. The study’s findings are consistent with the proposition that the OSCC associated with OSMF lesions develop through a specific intracellular signaling pathway associated with areca nut induced pathogenesis, which accounts for the different prognosis between OSCC associated with OSMF and OSCC without OSMF. This might contribute to the better grade of tumor differentiation (Fig. 3), lower likelihood of nodal metastases along with early detection (early clinical TNM stage) which shows better prognosis and the prevalence in younger males [9].

On observation of difference in clinical presentation amongst OSCC without OSMF and OSCC associated with OSMF, significantly high number (72, 43.4%) of cases clinically presented as ulcero-endophytic in OSCC without OSMF. Whereas in OSCC associated with OSMF, significantly high number of cases clinically presented as nearly equal number of ulcero-proliferative (55, 35.7%) and proliferative-exophytic (50, 32.5%). Although no study has revealed the difference in clinical presentation amongst OSCC without OSMF and OSCC associated with OSMF, the varied site specific clinical presentation was observed in OSCC. We observed buccal mucosa is the most common site affected by the OSCC associated with OSMF where as in OSCC without OSMF gingivo-buccal sulcus was commonly affected. Although the lateral border of tongue is considered as the most common site for OSCC in America and Europe, the buccal mucosa is the most common site for OSCC in southeastern Asia, due to the habits of areca nut and tobacco-chewing [18]. In OSMF there is initial involvement of buccal mucosa by fibrosis due to injudicious use of areca nut, which further involves the other areas of the oral mucosa like labial mucosa, palatal mucosa, and faucial pillars [19]. The leukoplakic, and erythroplakic lesions are frequently presented in the buccal mucosa, especially in close proximity to gingivo buccal sulcus anteriorly. However in the subsequent posterior aspect of the buccal mucosa it is more often secondary to trauma or lichen planus like lesions [20]. Usually erythroplakic lesions which shows induration, eventually take on an exophytic appearance. Our own observations revealed considerable attrition and uneven rough edges in OSMF patients’ posterior teeth. Along with these attributable functional restrictions owing to fibrosis in OSMF may results in ulcerations of oral mucosa which further with constant trauma may lead to tissue proliferation.

Yi-Ping Wang observed that about 90% of OVH lesions ensued mostly in patients with betel quid and areca nut chewing habits and in Taiwan buccal mucosa was the most frequently affected site in areca nut quid chewing-related oral cancers. This site predilection also suggests the close association of OVH lesions with areca nut quid chewing habits [21]. Jayasinghe LA et al. observed a wide spectrum of clinical presentations in both OSMF and OSCC originating from it. There was no evidence of invasion in histopathologically observed tissues in study group of patients who clinically presented with large verrucous lesions with characteristics of malignant lesion. This adverse histopathological interpretation may subject the patients for repeat tissue biopsies based on the assumption that the biopsy has been inadequate or nonrepresentative. These exophytic lesions in case of OSMF provide definitive indications for a clinician to arrive at the clinical diagnosis of OSCC. OSMF is distinct from all other OPMDs, because the main pathology is in the connective tissue, which is densely fibrosed. Consequently the connective tissue, which has abnormal cross-linking of collagen, may be impervious to the invasive progression in the connective tissue and thus letting further upward proliferation of tissue which results to an exophytic growth [15]. Further, the normal matrix metalloproteinases may not be effective in destroying the abnormal collagen to enhance the process of invasion [22].

This study is limited by its single-centre design. Future research should include larger, multicenter studies with prolonged follow-up and broader molecular profiling, in order to gain a better understanding into the prognosis of OSCC. In the present study, the causal relationships between clinical presentation and oncological outcomes cannot be established due to retrospective study design. Similarly, no longitudinal monitoring of clinical transformation such as the progression of OPMDs to OSCC over time, which could provide stronger evidence for early proliferative clinical patterns being predictive of malignancy in OSMF patients. Thus, future cohort studies are recommended on OPMD’s with long term follow up for any early clinical changes suggestive of malignant transformation.

## Conclusion

Many results from the current investigation, which included participants with histopathologically confirmed OSCCs, supported the previously proposed distinctive features of OSCC associated with OSMF that is better prognosis and oncological outcomes as well as a superior clinicopathological profile. A significantly higher number of OSCC cases associated with OSMF were clinically presented as ulcero-proliferative and proliferative exophytic lesions. Thus early detection is an important prerequisite for better prognosis of OSCC patients which relates favorably to OSMF patients. The functional limitations experienced by OSMF patients often lead to their early clinical presentation. In addition to its characteristic clinical presentation the proliferative, exophytic nature of the lesion may also contribute in early detection and diagnosis by providing considerable period for recognition and treatment planning and further surgical excision. Conversely, individuals with OSCC without OSMF typically do not experience pain or discomfort until the lesion progresses a stage that limits their functional considerations. Nevertheless, in order to better understand and support the findings, it is required to do a prospective analysis.

## Data Availability

The datasets used and/or analysed in the current study are available from thecorresponding author upon reasonable request.

## Abbreviations

OSCC: Oral squamous cell carcinoma
OSMF: Oral submucous fibrosis
OVH: Oral verrucous hyperplasia
OPMDs: Oral potentially malignant disorders
DMIMS (DU) IEC: Datta Meghe Institute of Medical Sciences (Deemed to be University) Institutional Ethical Committee
Tukey HSD: Honestly Significant Difference
SPSS: Statistics software
